# Ertapenem Articulating Spacer for the Treatment of Polymicrobial Total Knee Arthroplasty Infection

**DOI:** 10.1155/2016/5753489

**Published:** 2016-05-30

**Authors:** Dragan Radoicic, Milomir Milanovic, Jugoslav Marinkovic, Danica Radoicic

**Affiliations:** ^1^Orthopedic Surgery and Traumatology Clinic, Military Medical Academy, Crnotravska 17, 11000 Belgrade, Serbia; ^2^Clinic for Infectious and Tropical Diseases, Military Medical Academy, Crnotravska 17, 11000 Belgrade, Serbia

## Abstract

*Introduction*. Periprosthetic joint infections (PJIs) are the primary cause of early failure of the total knee arthroplasty (TKA). Polymicrobial TKA infections are often associated with a higher risk of treatment failure. The aim of the study was to assess the efficacy of ertapenem loaded spacers in the treatment of polymicrobial PJI.* Methods*. There were 18 patients enrolled; nine patients with polymicrobial PJI treated with ertapenem loaded articulating spacers were compared to the group of 9 patients treated with vancomycin or ceftazidime loaded spacers.* Results*. Successful reimplantation with revision implants was possible in 66.67%. Ertapenem spacers were used in 6 cases in primary two-stage procedure and in 3 cases in secondary spacer exchange. Successful infection eradication was achieved in all cases; final reimplantation with revision knee arthroplasty implants was possible in 6 cases.* Conclusion*. Ertapenem can be successfully used as antimicrobial addition to the cement spacers in two-stage revision treatment of polymicrobial PJIs. However, this type of spacer may also be useful in the treatment of infections caused by monomicrobial extended spectrum beta-lactamases producing gram-negative bacilli. Further clinical studies are required to evaluate the efficacy and safety of ertapenem spacers in the treatment of polymicrobial and monomicrobial PJIs.

## 1. Introduction

Periprosthetic joint infections (PJIs) are the primary cause of early failure of the total knee arthroplasty (TKA). In several large series of total knee arthroplasties, the prevalence of PJIs following TKA, in specialized centers, varies from 1.6% to 2.6% [1]. Given the current demographic prospects and increasing number of TKAs performed annually, the increasing longevity of these implants, and the longer life expectancies of patients, the prevalence of infections is expected to increase, as well as the medical and economic burden of infected TKAs [1, 2].

There are numerous organisms that can cause this devastating complication, but few of them are involved in the majority of knee PJIs.* Staphylococcus aureus* and coagulase-negative* Staphylococci *contribute to between 50 and 60% of PJIs, while* Streptococci *and* Enterococci *together account for only approximately 10% of cases [3, 4]. However, it is worth noting that gram-negative bacilli are isolated in 10% of cases of PJIs, and these infections are frequently polymicrobial [5].

In the abundance of published data on the subject of PJI, polymicrobial infections do not seem to get adequate attention, though when compared to monomicrobial PJI, polymicrobial infections are often perceived as more challenging to manage and associated with a higher risk of the treatment failure [6, 7].

Polymicrobial PJIs occur in up to 35% of early-onset infections, compared to <20% of infections occurring at any time point after arthroplasty implantation [3, 7]. One series found that 56% of all polymicrobial PJIs occurred within the first 90 days of implantation, compared to only 29% of monomicrobial PJIs,* Enterococcus* species,* S. aureus*, and aerobic gram-negative bacilli, including* P. aeruginosa*, which are the most frequently isolated bacteria, with each being present in more than one-quarter of infections [3, 7].

Antibiotic-loaded acrylic bone cement spacers are routinely used in the treatment of PJI. There are a limited number of antibiotics that have adequate elution kinetics, thermal stability, and mechanical properties that can be used with poly-methyl-methacrylate (PMMA) cements. The most commonly used and most extensively studied (in vitro and in vivo) are vancomycin, tobramycin, gentamycin, and clindamycin.

The aim of this study was to assess the efficacy of the ertapenem loaded articulating spacers in two-stage revision for polymicrobial TKA infection. To our knowledge this is the first article on the use of spacers loaded with ertapenem in polymicrobial prosthetic knee infections.

## 2. Methods

The study was conducted at University Center, Orthopedic Surgery and Traumatology Clinic. The clinic has 72 beds and three operating theaters and is a referral centre for primary and revision joint arthroplasty surgery. We retrospectively reviewed and acquired data on all the patients who underwent two-stage revisions for TKA infections. Operations were conducted at our institution during a period of 5 years (from 2010 to 2014).

For the purpose of this study we have only reviewed polymicrobial TKA infections as we have only used spacers loaded with ertapenem in this type of infections. Age, gender, comorbidities, American Society of Anesthesiologists (ASA) score, time of primary and revision surgery, microbiological culture results, dosage and duration of antimicrobial therapy, and antibiotics added to spacers were recorded. PJI was defined as the growth of the same microorganism in two or more cultures of synovial fluid or periprosthetic tissue, purulent synovial fluid or purulence at the implant site, acute inflammation upon histopathological examination of the periprosthetic tissue, or the presence of a sinus tract communicating with the prosthesis [8, 9]. Polymicrobial PJIs could be defined in the same manner only using the plural form for microorganisms.

The outcome of two-stage revision was classified as successful reimplantation or persistent infection. Persistent infections required additional surgery (knee arthrodesis or amputation).

Treatment was considered successful if the patient, after definitive reimplantation, had no symptoms or signs of infection and C-reactive protein (CRP), leukocyte count, and sedimentation rate were normal at the end of follow-up. Treatment was considered to have failed if the infection persistence did not allow the reimplantation or if the reimplanted prosthesis was removed because of the infection recurrence. The end of follow-up was defined as the last control visit concerning the treated prosthetic joint infection.

In brief, two-stage exchange involves resection of the implants, meticulous debridement and irrigation, placement of a temporary antibiotic-impregnated cement spacer, and delayed component reimplantation [10]. In all two-stage revisions we used antibiotic-loaded bone cement Refobacin Revision (Biomet, Warsaw, Indiana) already loaded with gentamycin and clindamycin. In most of the cases, to the abovementioned revision bone cement, additional antibiotics were added (2 g vancomycin, 4 g ertapenem, and 2–4 g ceftazidime).

Wounds were closed primarily, and no drains were used at any time of two-stage revision process. Intravenous (IV) antibiotics were started after intraoperative cultures were obtained. IV antibiotics were prescribed by infectious disease (ID) specialists (members of our institution orthopedic infections surveillance and treatment team) with adjustments made once the bacterial cultures and their antibiograms were available. After first stage, component removal and articulating spacer implantation, IV antibiotic therapy was continued for 2 to 4 weeks postoperatively, and most of the patients (where it was possible and made sense according to antibiograms) were on the oral antibiotics for 2 to 4 weeks afterwards. Some patients required repetition of the procedure, second spacer exchange, prior to definitive reimplantation, and in some cases reimplantation could not be carried out.

The antibiotic-free period before reimplantation was at least 6, up to 18 weeks, and if no symptoms or clinical findings of infection were present, second-stage reimplantation surgery was performed. Mandatory new tissue samples and cultures were obtained during this step, throughout debridement and spacer removal and before reimplantation of the new components. Successful reimplantations were performed with revision implants (NexGen LCCK—legacy constrained condylar knee and NexGen RH—rotating hinge knee prosthesis by Zimmer, Warsaw, Indiana).

IV antibiotics, preoperatively planned by ID specialists, were started after the specimens were taken. This antibiotic therapy was continued until new culture results were available.

If the results of microbiological cultures were positive, the antimicrobial treatment was changed according to antibiograms. If the bacteria were the same as in the primary infection, the PJI was classified as persistent infection. If the cultures were positive with some other microorganisms, the patient was treated as a case of new early PJI.

Patients with polymicrobial PJI treated with ertapenem loaded articulating spacers were compared to the group of patients treated with vancomycin or ceftazidime loaded spacers.

Due to small series only descriptive statistics were used.

## 3. Results

In the five-year period (2010 to 2014) we have identified and treated operatively with two-stage revision 49 infected TKAs. Primary TKA was performed at our clinic in 28 cases (during the researched period overall institutional TKA infection incidence was 2.8%), and 21 PJIs were referred from other institutions.

Polymicrobial TKA infections were present in 18 cases (36.73%). Monobacterial TKA infections were present in 31 cases. Mean age in the polymicrobial group was 66.61 and mean ASA score was 2.33, and there were 10 women and 8 men.

Polymicrobial infection was confirmed prior to the staged procedure in 12 cases. In six cases with preexchange confirmed polimycrobial infection we used articulating spacers, primarily loaded with 4 grams of ertapenem; in the other 2 grams of vancomycin were added to Refobacin cement. Handmixing of cement and antibiotic powder in a bowl without a vacuum was used in all cases, since bubbles facilitate elution of antibiotics by increasing surface area [11]. In all six cases of polymicrobial TKA PJI primarily treated with ertapenem spacers infection has subsided in the 2- to 4-week period, with clinical and laboratory signs of infection regression including sinus tract resolution. In 4 cases, final step in two-stage revision was performed with LCCK or RH knee Zimmer NexGen implants; in two cases, due to extensive bone loss and poor soft tissue coverage (in one case destroyed extensor mechanism), after removal of articulating spacers, knee arthrodesis was performed with bridging (across knee) external fixation.

Polymicrobial nature of TKA infection was established after debridement and obtaining of the tissue samples during articulating spacer implantation procedure (by intraoperative swabs and multiple tissue cultures) in 6 cases. In all those cases, after the first procedure there was evident infection persistence that required second two-stage procedure (reimplantation of antibiotic-loaded spacer) and ertapenem loaded spacers were used in three cases. In most of the polymicrobial infections gram-negative bacteria were involved (detailed data in Table 1).

In other six cases, polymicrobial nature of PJI was also confirmed before exchange; in 4 cases, to the Refobacin was added 2 g of vancomycin, in one case ceftazidime, and in one no additional antibiotics were added to the Refobacin cement. Of those patients undergoing a planned two-stage exchange, a successful reimplantation was performed in 2 cases; in one case as a final treatment, above knee amputation was performed. The other 3 patients with still active PJI underwent second two-stage procedure; ertapenem spacers were applied in the second two-stage exchange of spacers in two cases, and the treatment was successful. In one case the same spacer antibiotic combination was used (Refobacin Revision + 2 g vancomycin) but the infection persisted; arthrodesis was suggested; however, the patient refused and afterwards was lost to follow-up.

In total, successful reimplantation with revision implants was possible in 12 of 18 cases (66.67%). Ertapenem spacers, as treatment modality for polymicrobial TKA infections, were used in 6 cases in primary second-stage procedure and in 3 cases in secondary spacer exchange. Successful infection eradication was achieved in all cases and final reimplantation with revision knee arthroplasty implants was possible in 6 cases. When vancomycin loaded spacers were used for polymicrobial PJIs, reimplantation, after one spacer exchange, was possible in 3 cases.

## 4. Discussion

The therapeutic success of the ertapenem loaded spacers was confirmed with successful infection eradication and prosthesis reimplantation in all cases primarily treated with this new treatment strategy. The efficacy was additionally demonstrated with successful implementation in cases of polymicrobial PJIs primarily treated with other antibiotic spacer options.

There are several current surgical options for PJIs surgical treatment, irrigation and debridement (I&D) with component retention and one-stage and two-stage revision. Two-stage revision remains the most widely used approach, and many studies report high rates of successful (more than 90%) infection eradication [10, 12].

Although some authors report the shift from two-stage to one-stage revisions and I&D, based on reported rates of success, there is emerging consensus that two-stage exchange should be utilized in the more difficult patients, that is, those with significant comorbidities, resistant bacteria, or compromised wounds [13]. We believe that two-stage approach should be the treatment of choice and utilized in all cases of preoperatively confirmed polymicrobial PJI as well.

Traditionally, PJIs due to gram-negative organisms, though less common, are considered more difficult to manage, and the same could be said for the polymicrobial PJIs [14]. The reported rates of polymicrobial PJI range from 14 to 36% [15]. The diagnosis of polymicrobial PJI and identification of causative microbes is crucial for the adequate treatment planning, since PJIs of polymicrobial origin have been shown to have poorer clinical outcome than monomicrobial infections [16, 17]. The insufficient diagnosis of a polymicrobial infection can result in an insufficient postoperative antibiotic therapy and ultimately result in failure of the revised TKA. This can be avoided through the utilization of sonication, a relatively new diagnostic tool that has gained popularity in the last decade. Sonication leads to a significantly higher rate of bacterial isolation and a higher detection rate of polymicrobial isolations [16].

In the event of polymicrobial PJIs, especially when resistant bacteria are involved, there is evident need for more potent cement antibiotic combinations.

Ertapenem is a relatively new carbapenem developed to address the pharmacokinetic shortcomings (short half-life) of imipenem and meropenem, demonstrates broad-spectrum antimicrobial activity against many gram-positive and gram-negative aerobes and anaerobes, and is resistant to nearly all beta-lactamases, including extended spectrum beta-lactamases and AmpCs [18].

The degree of penetration of an antibiotic into the infection site is an important criterion for therapeutic success, which is particularly true during the treatment of osteoarticular infections. Applied parenterally, ertapenem has good diffusion into bone and synovial tissue [19]. The concentrations of ertapenem achieved in cancellous and cortical bone tissue and in synovial tissue were greater than the MIC90s for most aerobic organisms for 24 h and for 12 to 24 h for anaerobic bacteria in healthy volunteers undergoing total hip replacement [19].

On the other hand, there are scant published data available concerning the use of ertapenem in cement spacers for the PJI treatment. It has been shown that ertapenem meets the criteria for successful mixing with PMMA cements, though in vitro ertapenem does not sustain high elution rates of vancomycin and gentamycin, and they decrease from day 4 [20].

It seems that the goal to generate high local concentrations of antibiotic without associated systemic toxicity can be achieved with 4 g of ertapenem per 40 g of cement. The highest elution of antibiotics occurs within the first 24 to 72 hours followed by a prolonged release over several weeks, which correlates with the concentration of antibiotics within the cement itself [21, 22]; so the abovementioned ertapenem elution decrease from the day 4 does not significantly affect the therapeutic antibiotic effect.

This research is limited by its retrospective nature and small sample size.

Ertapenem can be successfully used as antimicrobial addition to the cement spacers in two-stage revision treatment of polymicrobial PJIs. However, this type of spacer may also be useful in the treatment of infections caused by monomicrobial extended spectrum beta-lactamases producing gram-negative bacilli. Further clinical studies are required to evaluate the efficacy and safety of ertapenem spacers in the treatment of polymicrobial and monomicrobial PJIs.

## Figures and Tables

**Table 1:** Polymicrobial TKA infections: patient characteristics and outcomes.

Patient age/gender	ASA score	Comorbidities	Microbes	First spacer exchange antibiotics	Second spacer antibiotics (if performed)	Outcome
68/female	2	Diabetes and hypothyroidism	*Staphylococcus aureus* and *Acinetobacter* spp.	Vancomycin 2 g + Refobacin		Reimplantation LCCK

57/male	3	Old heart attack and obesity	Methicillin-resistant *S. aureus Pseudomonas aeruginosa*	Vancomycin 2 g + Refobacin	Ertapenem 4 g + Refobacin	Reimplantation, RH Knee

59/female	2	Controlled hypertension and diabetes	*Klebsiella pneumoniae Serratia marcescens*	Refobacin		Reimplantation LCCK

73/female	2	Rheumatoid arthritis	*Enterococcus faecalis Staphylococcus epidermidis*	Ertapenem 4 g + Refobacin		Reimplantation LCCK

77/female	3	Hypothyroidism Stable angina	*Streptococcus* spp. *Serratia marcescens*	Ertapenem 4 g + Refobacin		Reimplantation LCCK

63/female	4	Unstable angina and renal failure	Methicillin-resistant *S. aureus Escherichia coli*	Vancomycin 2 g + Refobacin	Ertapenem 4 g + Refobacin	Reimplantation, RH Knee

72/male	2	Obesity and hypertension	*Klebsiella* spp. *Serratia marcescens*	Ertapenem 4 g + Refobacin		Reimplantation LCCK

76/male	1		*Staphylococcus aureus Peptostreptococcus* spp.	Vancomycin 2 g + Refobacin	Vancomycin 2 g + Refobacin	Knee arthrodesis

62/female	2	Rheumatoid arthritis, diabetes, and hypertension	*Klebsiella* spp. *Pseudomonas aeruginosa*	Ertapenem 4 g + Refobacin		Reimplantation LCCK

64/male	1		Methicillin-resistant *Staphylococcus aureus Pseudomonas aeruginosa*	Vancomycin 2 g + Refobacin		Knee arthrodesis

71/female	3	Controlled congestive heart failure and obesity	*Staphylococcus aureus Staphylococcus epidermidis*	Vancomycin 2 g + Refobacin		Reimplantation, RH Knee

55/female	4	Congestive heart failure and signs of hepatorenal failure	*Staphylococcus aureus Enterococcus*	Vancomycin 2 g + Refobacin	Vancomycin 2 g + Refobacin	Persistent infection. Lost to follow-up

72/male	1		*Streptococcus* spp. *Escherichia coli*	Ceftazidime 2 g + Refobacin		Reimplantation LCCK

69/female	2	Hypertension	Methicillin-resistant *S. aureus Enterococcus faecalis*	Vancomycin 2 g + Refobacin		Reimplantation LCCK

67/female	2	Diabetes	Coagulase-negative *Staphylococcus aureus* and *Proteus mirabilis*	Vancomycin 2 g + Refobacin	Ceftazidime 2 g + Refobacin	Persistent infection. Above knee amputation

59/female	3	Rheumatoid arthritis	Coagulase-negative. *Staphylococcus aureus*, *Klebsiella pneumoniae*	Vancomycin 2 g + Refobacin	Ertapenem 4 g + Refobacin	Reimplantation, RH Knee

72/female	4	Unstable angina and signs of hepatorenal failure	Methicillin-resistant Coagulase-negative *Staphylococcus aureus Escherichia coli*	Ertapenem 4 g + Refobacin		Knee arthrodesis

63/male	1		*Acinetobacter* spp. *Pseudomonas aeruginosa*	Ertapenem 4 g + Refobacin		Knee arthrodesis
